# The Isolation and Construction of an Infectious Clone for a Duck Adenovirus Type 3 Strain

**DOI:** 10.3390/microorganisms13061319

**Published:** 2025-06-05

**Authors:** Haipeng Lu, Mei Tang, Zhifei Zhang, Mi Wu, Chunxiu Yuan, Xue Pan, Qinfang Liu, Qiaoyang Teng, Bangfeng Xu, Minghao Yan, Dawei Yan, Fenglong Wang, Zejun Li

**Affiliations:** 1 College of Veterinary Medicine, Inner Mongolia Agricultural University, Hohhot 010010, China; 15024926734@163.com (H.L.); wumiregina@outlook.com (M.W.); lizejun@shvri.ac.cn (Z.L.); 2 Department of Avian Infectious Diseases, Shanghai Veterinary Research Institute, Chinese Academy of Agricultural Sciences, Shanghai 200241, China; 18773546303@163.com (M.T.); nzhangzhifei@163.com (Z.Z.); yuanchx@shvri.ac.cn (C.Y.); panxue@shvri.ac.cn (X.P.); liuqinfang@shvri.ac.cn (Q.L.); tengqy@shvri.ac.cn (Q.T.); xubangfeng@shvri.ac.cn (B.X.); m17519478956@163.com (M.Y.)

**Keywords:** duck adenovirus type 3 (DAdV-3), infectious clone, Hexon gene, biological characteristics

## Abstract

A duck adenovirus type 3 strain, SD2019, was isolated from sick Muscovy ducks in our laboratory in 2019. To study the biological properties of the virus, an infectious clone of the SD2019 strain was successfully established. The plasmid containing the whole genome of DAdV-3 was digested with *Pac* I and the linearized DNAs were electortransfected into LMH cells; the cells showed cytopathic effects (CPEs) at 96 h post transfection and the rescued virus (rSD2019) was identified by PCR and indirect immunofluorescence assays (IFAs). The biological characteristics of strain rSD2019 were studied in vitro and in vivo and the results show that rSD2019 grew to similar titers as compared with the wild-type SD2019 strain in LMH cells, as well as showing similar replication and virulence in Muscovy ducks. The establishment of a reliable reverse genetics system for DAdV-3 provides a foundation for future studies of DAdV-3.

## 1. Introduction

The *Adenoviridae* family comprises a diverse group of viruses capable of infecting various vertebrates, including mammals, birds, fish, and reptiles [1]. According to the 2023 International Committee on Taxonomy of Viruses (ICTV) classification, this family is divided into six genera: *Aviadenovirus*, *Barthadenovirus* (formerly *Atadenovirus*), *Ichtadenovirus*, *Mastadenovirus*, *Siadenovirus*, and *Testadenovirus*. Among these, *Aviadenovirus*, *Barthadenovirus*, and *Siadenovirus* are known to infect avian species, including chickens, turkeys, ducks, and geese.

Duck adenoviruses (DAdVs) are classified into two species: DAdV-A (also termed DAdV-1) and DAdV-B (comprising DAdV-2 to DAdV-6, with DAdV-6 being a proposed classification). While DAdV-A typically causes mild or asymptomatic infections in ducks, it can cross species barriers, infecting chickens and leading to Egg Drop Syndrome (EDS) [2,3]. In contrast, DAdV-B includes strains such as DAdV-2, DAdV-3, DAdV-4, and DAdV-5 (previously misclassified as fowl adenovirus 5), which share phylogenetic similarities with goose adenovirus 4 (GoAdV-4) (ICTV Global Report). Notably, DAdV-2 and DAdV-3, both isolated from Muscovy ducks, exhibit distinct genetic and pathogenic profiles. DAdV-2 possesses a single fiber gene and induces only mild symptoms, whereas DAdV-3 carries two fiber genes (fiber1 and fiber2) and demonstrates heightened virulence, causing severe hepatic and renal damage, including liver enlargement, hemorrhagic mottling, and kidney swelling [4]. Although DAdV-4 has been identified in recent years, it has not been associated with significant disease outbreaks [5].

Since its initial detection in 2014 [6], DAdV-3 has emerged as a major threat to Muscovy duck farms across China, including provinces such as Guangdong, Fujian, Zhejiang, and Anhui [7,8,9]. Outbreaks are characterized by high morbidity (40–55%) and mortality (35–43%) [10], leading to substantial economic losses in the duck industry. The pathogenic mechanisms underlying DAdV-3’s heightened virulence remain unclear, necessitating further investigation.

Reverse genetics systems of fowl adenoviruses have proven invaluable in studying viral pathogenesis, gene function, and host interactions. Successful applications in fowl adenoviruses—such as FAdV-1 (the CELO strain) [11], DAdV-3 [12], FAdV-4 [13,14,15], FAdV-8 [16], and FAdV-9 (the A-2A strain) [17]—have enabled genome manipulation either through direct in vitro modification or via infectious clones (e.g., FAdmids) generated by homologous recombination in *Escherichia coli*.

Here, a DAdV-3 strain SD2019 was isolated and identified from a Muscovy duck farm, we developed a reverse genetics system for DAdV-3, and the rescued virus was characterized in Muscovy ducks.

## 2. Materials and Methods

### 2.1. Cells, Plasmids, and Reagents

Leghom male hepatocellular (LMH) cells were purchased from the American Type Culture Collection (ATCC) and cultured in Dulbecco’s modified Eagle’s medium: F12 (DMEM/F12) (Hyclone, Logan, UT, USA), supplemented with 10% fetal bovine serum (FBS) (Biowest, South America origin, Riverside, MO, USA), 100 U/mL of penicillin, and 100 μg/mL of streptomycin (Sangon Biotech (Shanghai) Co., Ltd., Shanghai, China) at 37 °C with 5% CO_2_. DH5α-competent cells were purchased from Shanghai Weidi Biotechnology Co., Ltd. The plasmid PET28a (Sangon Biotech (Shanghai) Co., Ltd., Shanghai, China) was engineered to include the kanamycin resistance (Kana) gene. The DAdV-3 monoclonal antibody 2F12 was generated and stored in our laboratory. The FITC-labeled sheep anti-mouse IgG antibodies (Jackson, PA, USA) were purchased from Shanghai Nonin Biological Technology Co., Ltd., Shanghai, China. The DAdV-3 SD2019 strain was isolated from the livers of sick ducks in Shandong province and was passaged three times on the LMH cells by the limited dilution method, aliquoted, and stored at −80 °C.

### 2.2. (RT)-PCR Detection for Common Duck Viruses

The total RNAs and DNAs of the livers and kidneys of sick Muscovy ducks were extracted using the viral RNA/DNA purification kit (Tiangen Biotec (Beijing) Co., Ltd., Beijing, China) according to the manufacturer’s instructions. The RNAs were then reverse-transcribed into cDNAs following the manufacturer’s protocol (Tiangen, Beijing, China). To determine whether the samples were positive for common duck viruses, including duck parvovirus (DPV), goose parvovirus (GPV), duck enteritis virus (DEV), fowl adenovirus serotype 4 (FAdV-4), duck adenovirus serotype 3 (DAdV-3), duck circovirus (DuCV), avian influenza virus (AIV), duck Tembusu virus (DTMUV), duck hepatitis A virus type 1 (DHAV-1), duck hepatitis A virus type 3 (DHAV-3), Newcastle disease virus (NDV), and Novel duck reovirus (NDRV), the DNAs and cDNAs were used as templates for a PCR test (the primers are shown in Table 1).

### 2.3. Virus Isolation and Viral Amplification

The samples collected from the duck farm in Shandong province were weighed, and sterile PBS solution, supplemented with 1000 U/mL of penicillin and 1 mg/mL of streptomycin, was added at a ratio of 1 mg/mL. The samples were ground using a multi-sample tissue grinder at 180 Hz for 3 min after steel balls were added. The samples were subsequently centrifuged at 5000× *g* for 10 min and the supernatant was filtered through 0.22 µm filters. Exponentially growing LMH cells were seeded in a T25 cm^2^ flask. One day later, when the cells reached 90% confluence, 1.5 mL of DMEM/F12 containing 2% FBS was inoculated with the sample supernatant. After adsorption for 90 min at 37 °C, the supernatant was removed, and the cells were washed three times with 10 mM phosphate-buffered saline (PBS) and 5 mL of fresh DMEM-F12, supplemented with 1% antibiotic-antimycotic, and 2% FBS was added. When 70–80% of the LMH cells exhibited complete cytopathic effects (CPEs), the cells and supernatant were freeze–thawed three times and were then harvested. After the samples were centrifuged at 3000× *g* for 15 min at 4 °C, the supernatant was collected and stored at −80 °C. The viruses were purified three times in LMH cells using the limiting dilution method.

### 2.4. Phylogenetic Analysis

To investigate the evolutionary relationships of DAdV-3 SD2019 with other adenoviruses, the Hexon gene sequence of SD2019 was compared with those of 31 strains retrieved from the NCBI database, and the phylogenetic trees were constructed using the neighbor-joining (NJ) method in MEGA 11 software. The aligned Hexon gene sequences were analyzed to elucidate the phylogenetic relationships among these strains.

### 2.5. Construction of DAdV-3 Infectious Clone

The viral supernatant was ultra-centrifuged (100,000× *g*) in 30% sucrose in TNE buffer (10 mM Tris–HCl pH 7.5, 100 mM NaCl, 1 mM EDTA pH 8.0) at 4 °C for 2 h. The viral pellets were resuspended in TNE buffer followed by proteinase K treatment and DNA extraction with phenol, chloroform, and ethanol precipitation. A PCR product containing the ITR region, the *Pac* I cleavage site, the kanamycin-resistant gene and origin (KAN-ORI) were amplified with the primer pairs DAdV-3 PacI-F/R (Table 1). After gel purification, the KAN-ORI and DAdV-3 genomic DNA were included in a homologous recombination reaction containing the 2 μL of the DAdV-3 genome, 1 μL of KAN-ORI, 4 μL of 5× Exnase II buffer, 2 μL of Exnase II, and 11 μL of ddH_2_O to construct the DAdV-3 infectious clone pET-KanaR-DAdV-3 (pDAdV-3). After incubation at 37 °C for 30 min, the reaction solution was transformed into *E. coli* Top10 competent cells. Positive clones were further identified and verified using the primer pairs pDAdV-3 Kana F/R (Table 1).

### 2.6. Virus Rescue

Adenovirus plasmids including the whole DAdV-3 genome (pDAdV-3) were digested with the restriction enzyme *Pac* I (NEB, Ipswich, MA, USA), and the linearized DNAs were recovered by ethanol precipitation and then the DNAs were elector transfected into LMH cells (130 V, 950 μF,+∞, 2 mm). The mixed solution was transferred into a T25 flask with 5 mL of 37 °C-warmed DMEM/F12, supplemented with 10% FBS, and the supernatant was replaced with fresh DMEM-F12 containing 2% FBS after 24 h. The transfected cells were cultured at 37 °C with 5% CO_2_ for 6 days. The cells and the supernatant were freeze–thawed three times, when 80% transfected cells showed apparent cytopathic effects (CPEs), and the cellular debris was removed by centrifugation at 5000× *g* for 5 min. The supernatant was stored at −80 °C.

### 2.7. Indirect Immunofluorescence Assay (IFA)

LMH cells cultured on 6-well plates were infected with parental DAdV-3 SD2019 (wtSD2019) and rescued SD2019 (rSD2019) for the IFA test. Briefly, the LMH cells were inoculated with wtSD2019 or rSD2019 at an MOI of 0.01. At 72 hpi, the cell culture was removed and the infected cells were fixed with 4% paraformaldehyde for 20 min at room temperature (RT) and washed with PBS. The plates were blocked with 10% BSA for 20 min at RT and washed with PBS. Then the anti-DAdV-3 monoclonal antibody 2F12 (mAb, 1:200 dilution) was added into the wells and inoculated for 1 h at 37 °C. The wells were subsequently washed three times with PBS containing 1% Tween-20 (PBST) and incubated with 100 μL FITC-conjugated goat anti-mouse IgG antibody (1:400 dilution, Invitrogen, Carlsbad, CA, USA) for 1 h at 37 °C. After washing with PBS five times, the images of the cells were examined using a fluorescence microscope (Carl Zeiss, Oberkochen, Germany).

### 2.8. Viral Growth Kinetics

To test the replication of parental and rescued DAdV-3 SD2019 strains in vitro, LMH cells were infected with wtSD2019 and rSD2019 at an MOI of 0.01; the cells were incubated with DMEM/F12 containing 2% FBS at 37 °C for 2 h. After washing 3 times with PBS, the cells were cultured at 37 °C for 6 days and the viral supernatant was harvested every 12 h post infection and was subjected to virus titration on LMH cells to determine the virus titers.

### 2.9. Virus Titration

To assess the virus titers, the duck tissue and cell culture samples were titrated as previously described [3]. Briefly, weighed tissue samples were homogenized in sterile PBS to form 1:1 (mL/g) suspensions. The homogenates, along with cell culture samples, were centrifugated at 12,000× *g* for 10 min at 4 °C. Undiluted and 10-fold serially diluted supernatants were added onto the LMH cells in 96-well plates at 37 °C for 2 h. Following adsorption, the cells were washed by PBS and cultured in DMEM/F12 with 2% FBS at 37 °C with 5% CO_2_. The lower limit of virus detection was 0.5 log10 TCID_50_ per 0.1 g tissue, and titers were calculated using the Reed-Muench method.

### 2.10. Duck Experiments

To test the virulence of parental and rescued SD2019 in Muscovy ducks, eighteen 21-day-old Muscovy ducks were randomly divided into 3 groups. Ducks in two of the three groups were inoculated intramuscularly (i.m.) with 10^5.5^ TCID_50_ of each virus (wtSD2019 or rSD2019) at a volume of 0.2 mL, respectively. The ducks in another group were inoculated with an equal volume of sterile PBS. The ducks were monitored daily for clinical signs until 14 dpi. At 4 dpi, the liver and kidney samples of 3 ducks in each group were collected for a qPCR test, respectively. The sera of another 3 ducks in each group were collected at 0, 7, and 14 dpi for antibody detection.

### 2.11. The qPCR Assay

To quantify the DAdV-3 DNA copies in the duck tissue samples, a real-time qPCR assay was performed. The forward (5′-TGACATAAAGGGTGTGCTAG-3′) and reverse (5′-GGAGCTAGTGGATTGTAAG-3′) primers and probe (5′-FAM TCTTCTTTCAAACCATACAGTGG-BHQ1-3′) were designed based on the Hexon gene of DAdV-3 and were synthesized at Sangon Biotech (Shanghai) Co., Ltd. The tissue samples were homogenized in PBS at a ratio of 1:1 g/mL and centrifugated at 12,000× *g* for 10 min at 4 °C. The supernatant was used for DAdV-3 DNA quantification. Total DNAs were extracted using the Ezup Column Virus DNA purification Kit (Sangon Biotech, Shanghai, China). In total, 12.5 μL of 2× Multiplex Probe qPCR Mix Plus, 0.5 μL of Forward primer (10 μM), 0.5 μL of Reverse primer (10 μM), 1 μL of Probe (10 μM), 2 μL of DNA template, and 8.5 μL of ultrapure water were added into a DNAase and RNAase-free tube and the real-time qPCR was performed on a Mastercycler ep realplex system (Eppendorf, Hamburg, Germany) using the following cycling conditions: 37 °C, 2 min, 95 °C 30 s, (95 °C 10 s, 60 °C, 30 s) × 40 cycles.

### 2.12. Antibody Detection

A blocking ELISA for the detection of serum antibodies against DAdV-3 was developed and performed as previously described [18]. In summary, ELISA plates were coated with purified DAdV-3 antigen in 0.1 M carbonate–bicarbonate buffer (pH 9.6) and incubated overnight at 4 °C. The plates were washed with PBST (PBS with 0.05 % Tween-20), and blocked with 5 % skimmed milk dissolute in PBS for 1 h at 37 °C and then incubated with 1:10 diluted serum sample for 1 h at 37 °C. After washing, mAb 2F12 (1:1000) was added and incubated at 37 °C for 1 h, followed by HRP-labeled goat anti-mouse IgG (1:4000, Sigma, St.Louis, MO, USA) was added and incubated at 37 °C for another 1 h. The TMB substrate was added and incubated at room temperature for 10 min, then stopped with 0.1 N sulfuric acid. The optical density (OD) was measured at 450 nm, and the percent inhibition (PI) was calculated as PI (%) = [1 − (OD450 nm of test serum/OD450 nm of negative control serum)] × 100%. A PI value ≥21.62% indicated a positive result, while a value ≤16.79% indicated a negative result.

### 2.13. Ethics Statement and Statistical Analysis

All animal experiments were carried out in accordance with the recommendations in the Guide for the Care and Use of Laboratory Animals of the Ministry of Science and Technology of China. The protocol (SV-20240526-01) used in the study was approved by the Animal Care Committee of the Shanghai Veterinary Research Institute.

## 3. Results

### 3.1. Virus Isolation and Identification

**Figure 1:**
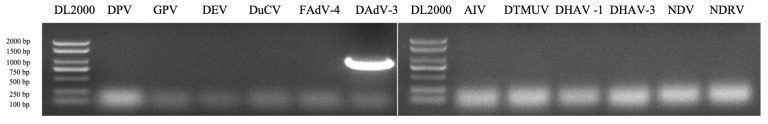
Tissue samples tested by RT-PCR and PCR for common duck viruses. The RT-PCR and PCR analysis revealed 1014 bp of the DAdV-3 target sequence in lane 7. Lanes 2 to 6 and lanes 9 to 14, which included DPV, GPV, DEV, DuCV, FAdV-4, AIV, DTMUV, DHAV-1, DHAV-3, NDV, and NDRV, showed no interference with the target sequence. Lanes 1 and 8 served as the DL2000 marker.

In 2019, an infectious disease outbreak characterized by swelling and hemorrhagic livers and kidneys occurred in a Muscovy duck farm in Shandong. The liver and kidney samples collected from two affected duck farms were homogenated and centrifuged; the supernatant was subjected to RT-PCR and PCR tests. The results show that the tissue samples were positive for DAdV-3 (Figure 1, but were negative for other common duck viruses, including DPV, GPV, DEV, DuCV, FAdV-4, AIV, DTMUV, DHAV-1, DHAV-3, NDV, and NDRV (Figure 1). To isolate the virus, the samples were inoculated into LMH cells and the infected LMH cells showed CPEs at 4 days post infection, which suggested that the virus is virulent in LMH cells. To purify the virus, the infected cells and the supernatant were harvested and freeze–thawed 3 times, the mixtures were centrifuged at 12,000× *g* at 4 °C, and the supernatant was purified 3 times in LMH cells by the limiting dilution method. A DAdV-3 strain, isolated and named SD2019, was further verified by sequencing and the sequence was submitted to GenBank.

### 3.2. Phylogenetic Analysis

**Figure 2:**
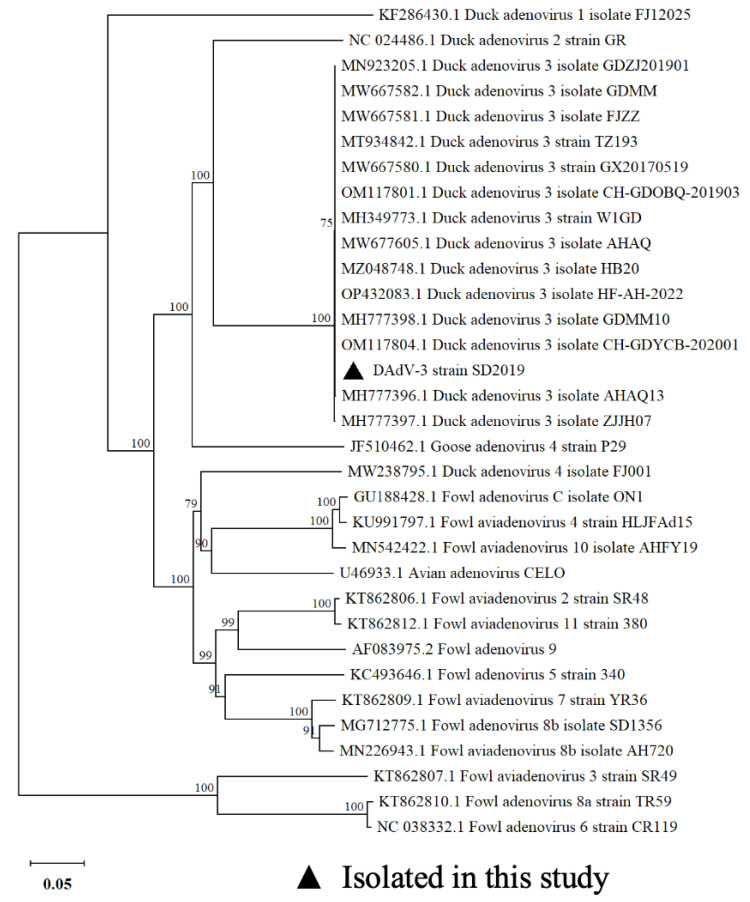
The phylogenetic analysis based on the Hexon gene of adenoviruses. The phylogenetic tree was generated using the NJ method based on the Hexon gene sequence in MEGA v.11.0. For each virus strain, GenBank accession numbers, strain types, and names are shown.

To verify the virus, the Hexon gene sequence of DAdV-3 SD2019 was amplified for sequencing. To determine the phylogenetic relationship of DAdV-3 SD2019 and other reported fowl adenoviruses, the Hexon genes, including DAdV-3 2019 and other 31 representative fowl adenoviruses, were used for further analysis. A phylogenetic tree was generated by using the neighbor-joining (NJ) method in MEGA 11.0 version software. The phylogenetic analysis showed that DAdV-3 SD2019 was grouped into DAdV-3, and shared a high level of sequence identity (98.5–100%) with other previously reported DAdV-3 strains (Figure 2).

### 3.3. Generation of DAdV-3 Infectious Clone

**Figure 3:**
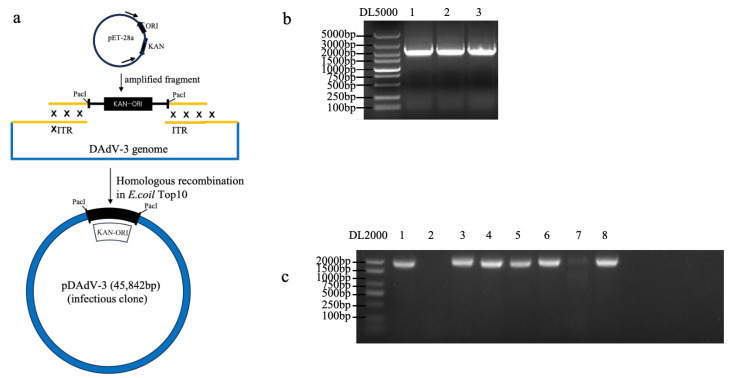
The generation of the infectious clone of DAdV-3. (**a**) A schematic diagram of the generation of the infectious clone. (**b**) PCR amplification for KAN-ORI. Lanes 1 to 3, 2200 bp of PCR products containing the ITR region. The *Pac* I cleavage site, kanamycin-resistant gene and origin (KAN-ORI) were amplified with the primer pairs DAdV-3 *Pac* I-F/R (Table 1). (**c**) The identification of positive clones by PCR. Clones 1, 3, 4, 5, 6, and 8 revealed 1800 bp of target sequence, and were identified as positive.

To generate the infectious clone of DAdV-3 (Figure 3a), the *Pac* I cleavage site, the kanamycin-resistant gene and origin (KAN-ORI) sequences were amplified (Figure 3b) and were recombined with viral DNAs. Then the plasmid was circularized through homologous recombination and transformed into *E. coli* Top10 competent cells. After 24 h of incubation, clones grown on kanamycin-resistant LB plates were screened via PCR for positive recombinants (Figure 3c). A plasmid containing the full-length DAdV-3 genome was successfully constructed through homologous recombination and designated pDAdV-3.

### 3.4. Virus Rescue

**Figure 4:**
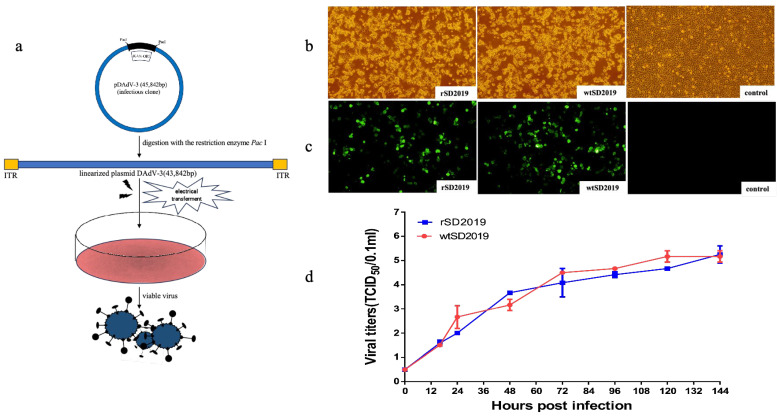
Virus rescue and validation. (**a**) A schematic diagram of the virus rescue. (**b**) The infected LMH cells showed cytopathologic effects characterized by cell congregation, contraction, rupturing, and detachment at 96 h post transfection. (**c**) The immunostaining of LMH cells infected with the rSD2019 and wtSD2019 at 72 h post inoculation. The infected cells were stained with MAb 2F12 and FITC-labeled goat anti-mouse IgG. (**d**) Viral growth curves. LMH cells were infected with rSD2019 or wtSD2019 at multiplicities of infection of 0.01 and the supernatant was collected at different time points and titrated on LMH cells. The virus titer data represent the means from three repeats, with error bars showing the standard errors.

To rescue DAdV-3 SD2019, the plasmids containing the whole genome of DAdV-3 SD2019 (pDAdV-3) were digested by the *Pac* I, and the linearized DNAs were then electrotransfected into LMH cells (Figure 4a). Detectable CPEs appeared at 96 h post transfection, while no CPEs were found in untransfected cells (Figure 4b). The transfected cells and supernatant were harvested and used for an IFA test. Fresh LMH cells infected with the rescued virus showed obvious specific green fluorescence at 72 h post infection, while no fluorescence was detected in uninfected cells (Figure 4c). The rescued viruses’ passage 2 (P2) were used for PCR testing and sequencing. The results show rDAdV-3 SD2019 P2 was positive for DAdV-3 and the sequence analysis confirmed the rescued virus has no unexpected mutations. The results indicated that the virus was successfully rescued.

### 3.5. Growth Curves of rSD2019 and wtSD2019 in LMH Cells

To determine the replication kinetics of rSD2019 and wtSD2019, LMH cells, cultured in T25 flasks, were inoculated with different viruses at multiplicities of infection of 0.01, respectively. The supernatant was collected at every 12 h post-infection and titrated. Compared with that of wtSD2019, the rSD2019 grew similarly within 144 h post-infection (Figure 4d).

### 3.6. Pathogenicity of rSD2019 and wtSD2019 in Muscovy Ducks

To evaluate and compare the pathogenesis of the rescued and parental SD2019 in ducks, six 21-day-old Muscovy ducks were inoculated intramuscularly with 10^5.5^ TCID_50_ of rSD2019 or wtSD2019, respectively. No ducks died during the entire experiment.

**Figure 5:**
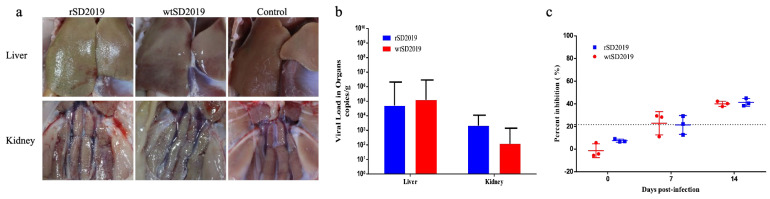
The pathogenicity of the rescued and parental SD2019 in Muscovy ducks. Six 21-day-old Muscovy ducks were inoculated with 105.5 TCID50 of rSD2019 or wtSD2019 intramuscularly. (**a**) The lesions of livers and kidneys of ducks inoculated with the rSD2019 and wtSD2019 at 4 dpi. (**b**) Viral DNA copies in livers and kidneys of ducks in the rSD2019 and wtSD2019 groups at 4 dpi. The data for viral DNA copies indicate the results for 3 ducks. (**c**) Antibodies generated against DAdV-3 in infected ducks were quantified by blocking ELISAs using serum obtained at 0, 7, and 14 dpi. The serum was considered positive when the PI value was ≥21.62%. The dashed line in the figure indicates the threshold value of 21.62% for determining seropositivity.

At 4 dpi, the livers and kidneys of infected ducks showed hemorrhage in both the rSD2019 and wtSD2019 groups (Figure 5a). Viral DNA copies in different tissue samples were quantified by real-time qPCR at 4 dpi and all the livers and kidneys tested were positive for DAdV-3 DNA. In the liver and kidney samples, the virus titers did not significantly differ between the rSD2019 and wtSD2019 groups, respectively (Figure 5b). Two ducks in three inoculated with either the parental wtSD20199 or its rescued strain rSD2019 showed seroconversion at 7 dpi. And the sera of the last one were positive in each group (Figure 5c). All the results indicate that rSD2019 replicates similarly compared to wtSD2019 in Muscovy ducks.

## 4. Discussion

The first isolation of a novel duck adenovirus type 3 (DAdV-3) strain from infected Muscovy ducks in China was reported in 2014 [6]. Since then, DAdV-3 infections have increasingly affected duck farms, emerging as a significant threat to the poultry industry with considerable economic impacts [7,8,9].

The pathogenicity of different DAdV-3 strains differs in Muscovy ducks. The DAdV-3 TZ193 strain caused characteristic lesions or swelling as well as hemorrhagic liver and kidney in the infected ducklings, and the mortality rate of TZ193 in 5-day-old domestic ducks was 100% [9]. FJGT01 was highly virulent for ten-day-old Muscovy ducklings, with a mortality rate of 60%, which started on day 5 after inoculation. Meanwhile, gross lesions showed signs of swelling and hemorrhage in the liver and the kidneys of infected ducklings [7]. The morbidity of CH-GD-12-2014 to one-day-old SPF Shaoxing ducks and Muscovy ducks is 100% for both, but the mortality to these two kinds of ducks differs; CH-GD-12-2014 caused 7 out of 25 one-day-old SPF Shaoxing ducks’ deaths, while no infected Muscovy ducks died [6]. In another study, DAdV-3 CH-GD-12-2014 caused the death of 10-week-old ducks [12]. Interestingly, HF-AH-2020 has a very low pathogenicity in Muscovy ducks, but can induce a high level of cellular immunity [4]. In this study, we isolated a wtDAdV-3 SD2019 strain on LMH cells, and the Hexon gene sequence shared 98.7–100% similarities with that of other DAdV-3 reference strains. Establishing a challenge model at an appropriate age for immunized ducks in the future should be considered; the 21-day-old Muscovy ducks were infected with the wtSD2019 strain. However, we found that wtSD2019 was not fatal to 21-day-old Muscovy ducks. Additionally, the lesions of the livers and kidneys seemed to be relatively mild at 4 dpi, which indicated that the wtSD2019 also has low pathogenicity to Muscovy ducks compared to some other reported strains.

To further study the mechanism of different virulence levels of DAdV-3 strains in waterfowl, a reverse genetics system for DAdV-3 should be established. Here, we have generated the DAdV-3 infectious clone and rescued the virus (rSD2019) successfully. The rescued virus exhibited identical CPE formation, immunological reactivity, and replication capacity to the wild-type virus, demonstrating both the reliability of our reverse genetics system and the preserved infectivity of the cloned genome. The reverse genetics system for DAdV-3 provides a powerful tool for studying viral pathogenesis [3,19] and developing vaccines [12,14,15]. While there are challenges associated with the complexity and biocontainment requirements, the system offers significant advantages in terms of precision, flexibility, and efficiency. Future research should focus on optimizing the system for vaccine development and exploring its potential applications in antiviral drug screening.

Infectious clones of various FAdV serotypes have been developed using diverse techniques, including recA-mediated recombination in *E. coli* BJ5183 [20], two-step Red/ET-mediated recombination [12,21], and λRed-mediated recombination in *E. coli* DH10B [22]. However, in vivo homologous recombination is inconvenient, requiring the co-transformation of inserts and vectors with homology arms, the recovery of recombined plasmids from cloned cells, and their subsequent re-transformation into expression cells [23]. In this study, an in vitro homologous recombination Exnase II cloning method was used. Exnase II, also known as the Exo-Beta recombinase or Exo-Beta system, is a highly efficient multi-enzyme recombination system. This highly efficient multi-enzyme system enables recombination with minimal homologous arms (15–25 bp), operates with low mismatch rates and high precision, and completes reactions in just 30 min at 37 °C. This approach facilitates rapid and efficient DNA assembly through exonuclease and polymerase-enhanced recombination [23,24].

In summary, a duck adenovirus type 3 strain SD2019 was isolated and an infectious clone, based on the full length of SD2019, was successfully established. The reverse genetics system developed here not only provides a robust platform for elucidating the pathogenic mechanisms underlying the differing virulence levels of DAdV-3 strains but also paves the way for the development of effective vaccines and antiviral strategies to improve disease control and prevention.

## Figures and Tables

**Table 1:** Primers used in the study.

Primer Name	Sequence (5′→3′)	Target Gene	Size
DAdV-3-F	GTACCGCCTTCGAGAACACA	Fiber 1	1014 bp
DAdV-3-1014R	GCTGTCTGATTCTGGTGATC		
DPV-REP-F	CAAACGGGGAGGGCAAAATAAGA	VP 2	600 bp
DPV-REP-600R	GTGGTCGCAGGTCCGTAGAGC		
GPV-F	CCAAGCTACAACAACCACATCTAC	VP 1	375 bp
GPV-375R	CTGCGGCAGGGCATAGACATCCGAC		
DEV-F	GGACAGCGTACCACAGATAA	UL30	520 bp
DEV-520R	ACAAATCCCAAGCGTAG		
DuCV-F	CGGCGCTTGTACTCCGTACTC	Rep	619 bp
DuCV-619R	CCCGCGTGGTTTGTAATACTTG		
FAdV-4-F	CAACTACATCGGGTTCAGGGATAACTTC	Hexon	667 bp
FAdV-4-667R	CCAGTTTCTGTGGTGGTTGAAGGGGTT		
AIV-F	TTCTAACCGAGGTCGAAAC	M	229 bp
AIV-229R	AAGCGTCTACGCTGCAGTCC		
DTMUV-F	CATAGGCTGGAATCTGGGAAC	E	300 bp
DTMUV-300R	TCTGGATTCTGTCGTCACGTC		
DHAV-1-F	CAGTTTACCGCCCCACTCTAT	VP 1	699 bp
DHAV-1-699R	TGGCTTCCACCTCCTCTTCAT		
DHAV-3-F	GAAATCTGCACTCAATGGAGAG	VP 1	286 bp
DHAV-3-286R	CCCAGGAAATGATTGGTCAG		
NDV-F	TGTAGTAACGGGAGACAAAGCAG	F	350 bp
NDV-350R	GAATAAATACCAGGAGACATAGGGA		
NDRV-F	GCATGAACATGCCAGTTGAG	S1	300 bp
NDRV-300R	AAGCCATAACGATGGCAGTC		
DAdV-3-PacI-F	CAATTTACCGATGGTATATATATGATGATGTTAATTAATTTCCATAGGCTCCGC		2200 bp
DAdV-3-PacI-2000R	CAATTTACCGATGGTATATATATGATGATGTTAATTAAACGCGCCCTGTAGCGG		
pDAdV-3 Kana F	AGCGTGGCGCTTTCTCATAG		1800 bp
pDAdV-3 Kana R	CTTTCGCTTTCTTCCCTTCC		

## Data Availability

The original contributions presented in this study are included in the article. Further inquiries can be directed to the corresponding authors.
